# Degenerative cervical myelopathy: timing of surgery

**DOI:** 10.1530/EOR-2025-0070

**Published:** 2025-06-02

**Authors:** Maria Rossella Fasinella, Alberto Benato, Donato Creatura, Alexis Morgado, Cédric Yves Barrey

**Affiliations:** ^1^ Department of Spine and Spinal cord surgery, Hôpital Pierre Wertheimer, GHE, Hospices Civils de Lyon, and Claude Bernard University of Lyon 1, Lyon, France; ^2^ Laboratory of Biomechanics, ENSAM, Arts et Metiers ParisTech, Paris, France

**Keywords:** degenerative cervical myelopathy (DCM), spinal cord compression, traumatic spine injury, surgical timing

## Abstract

**Background:**

- Despite the growing burden of degenerative cervical myelopathy (DCM), consensus on the optimal timing of surgical intervention remains lacking, especially for patients with mild symptoms or asymptomatic cord compression or in the context of recent trauma. Different scores, such as the mJOA, Nurick scale and NDI are commonly used to classify disease severity, but guidelines for managing these patients do not provide a clear framework for intervention timing.

**Materials and methods:**

- We conducted a narrative review of the literature on the optimal timing of surgical intervention for DCM, using PubMed to identify relevant studies. The search was focused on surgical and non-operative management, clinical and radiological assessments, biomarkers and emerging technologies. The selected papers were reviewed for relevance and quality, with guidance from a senior author.

**Results:**

- The initial search identified 6,705 articles, which were narrowed down to 136 relevant studies after applying filters for study type and clinical focus. A final selection of 87 papers was categorized by topics and the findings were synthesized to highlight trends, challenges and knowledge gaps in surgical timing for DCM.

**Focus of the study:**

- This review article examines strategies for determining the optimal timing for surgery in DCM. It explores how radiological signs, clinical indicators and other markers may help identify patients at risk of rapid neurological deterioration, particularly in the ‘grey-zone’ population (mild symptoms or asymptomatic disease), enabling clinicians to assess correctly different clinical scenarios and to indicate timely surgical intervention.

## Introduction

Degenerative cervical myelopathy (DCM) is a progressive spinal disorder and the leading cause of spinal cord dysfunction in adults worldwide (1). With the aging population, the prevalence of DCM is expected to rise, creating an escalating challenge for clinicians in treating patients with diverse levels of neurological impairment. The disease manifests along a wide spectrum, from mild sensory disturbances to severe motor deficits, often leading to significant and irreversible functional decline. Due to its progressive nature, early diagnosis and timely surgical intervention become essential to mitigating neurological deterioration and preventing irreversible spinal cord damage.

Despite the growing burden of DCM, consensus on the optimal timing of surgical intervention remains a subject of debate. Current classifications define disease severity using the modified Japanese Orthopedic Association (mJOA) score, categorizing patients as mild (15–17), moderate (12–14) or severe (≤11) (2). However, guidelines for managing these different patient groups – especially those with mild symptoms or asymptomatic cord compression – are not well established (1). In fact, surgery is the recommended treatment for moderate to severe cases, while uncertainty persists regarding whether early operative intervention benefits patients with milder disease. In addition, the role of surgery in very severe patients (mJOA ≤8) is still a matter of discussion.

Recent systematic reviews have sought to clarify these issues by addressing three key objectives: i) identifying patients at high risk of neurological deterioration, ii) evaluating the role of operative versus non-operative management, and iii) determining which patient populations derive the greatest benefit from surgical intervention (3); with the ultimate objective to define what is the optimal timing for surgery in DCM patients.

This review therefore examines strategies for determining the optimal timing for surgical intervention in DCM. We focused on the evidence that may aid in the identification of the moment when the so-called ‘grey-zone’ patients (mild symptoms or asymptomatic) are most at risk of experiencing rapid neurological deterioration, and thus avoid it through timely intervention.

## Materials and methods

In this study, we performed a narrative review of the literature to investigate the optimal timing of surgical intervention in DCM. Comprehensive research was conducted on the PubMed database by two authors (AB and MRF) using standardized keywords related to DCM, with surgical and nonoperative management, post-operative outcome predictive factors, radiological assessments, biomarkers predictive potential and new technologies applied to clinical evaluation and diagnosis. The resulting preliminary pool of articles was discussed with the senior author (CB) to identify the papers providing the most relevant and up-to-date information guiding decisions on the timing of surgery for DCM.

Data from the selected studies were synthesized and organized to identify trends, challenges and gaps in the current understanding of the optimal timing for surgical intervention for DCM, with a special focus on patients with early DCM and non-myelopathic patients.

## Results

The search conducted on PubMed, using the keywords ‘cervical myelopathy’ and applying filters for articles published in the last 15 years in English on human subjects, adults (≥18 years of age) and elderly (≥65 years of age), initially identified 6,705 articles. After narrowing the search to the abovementioned article types (meta-analyses, randomized controlled trials, systematic reviews, cohorts, case series, guidelines and recommendations, administrative databases and surveys), the number of articles dropped to 429, which were further analyzed for their relevance and methodological quality. By adding the keywords concerning the main topic of our research, namely the clinical-radiological aspects that help define surgical timing; the number of relevant articles was reduced to 136.

The final selection consisted of 87 papers, that have been categorized according to their main focus on: clinical assessment, guidelines, early intervention, natural history of DCM, nonoperative treatments for early DCM, radiological findings with relevance for surgical decisions, special factors with potential influence on surgical timing (OPLL, comorbidities and frailty, trauma, sagittal alignment and deformity), current surgical standards and future developments. The data discussed in the selected papers were organized and summarized according to their focus.

## Discussion

### Preoperative assessment and clinical scenarios

**Figure 1:**
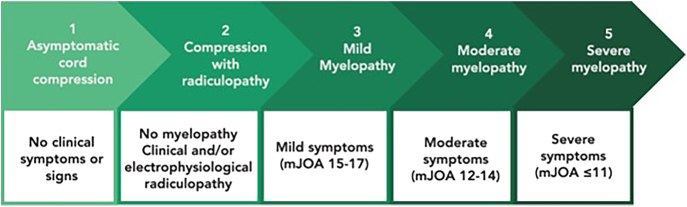
The five clinical categories described by Fehlings *et al.* in 2017 (62) are schematically illustrated above.

When discussing the topic of DCM, five main groups of patients can be identified (1, 2, 4, 5) (Fig. 1): i) asymptomatic patients with radiological evidence of spinal cord compression; ii) patients with clinical or electrophysiological evidence of radiculopathy and radiological cord compression; iii) patients with mild myelopathy (mJOA: 15–17); iv) patients with moderate myelopathy (mJOA: 12–14); and v) patients with severe myelopathy (mJOA: 11 or less).

The first two categories are collectively referred to as ‘non-myelopathic’ patients. However, this term can be misleading, as spinal cord injury may already be present, as evidenced by histopathology, biomarkers (6) or advanced imaging and neurophysiology techniques (4, 7, 8). Furthermore, alternative terms such as ‘asymptomatic degenerative spinal cord compression’ (4) exclude patients with radiculopathy and/or neck pain, further complicating classification.

Asymptomatic spinal cord compression (ASCC) (category 1) is a common condition in the aging population, with prevalence estimates ranging from 40% to 60% among older Western adults (4, 5). A meta-analysis estimated a global prevalence of 24.2% (9), with significantly higher rates in older Western populations and lower rates in Asian populations (9).

Among non-myelopathic patients, it is crucial to distinguish those with cervical radiculopathy, as some evidence suggests a significantly higher risk of progression to clinical myelopathy (1, 4, 5). Due to this increased risk, international guidelines discuss this group separately (1, 10). Electrophysiological evidence of radiculopathy has been associated with an increased risk of myelopathy development as well (1, 11). In the case of chronic radicular signs on EMG but without clinical radicular symptoms, the patient should then be considered as part of category 2. In addition to radiculopathy, neck and shoulder pain are important symptoms to recognize, as they may be the only presenting complaints. Such cases should prompt clinicians to search for more subtle signs of DCM (12, 13).

The prevalence of symptomatic DCM is estimated at 2.3% in the general population smith 2021. A minority of patients progress from ASCC (group 1) to clinical myelopathy (groups 3–5), making them potential candidates for surgery. However, clinical progression can be subtle, emphasizing the need for effective diagnostic tools to prevent delays. Studies indicate that DCM diagnosis is often delayed by 1.5 to over 2 years from symptom onset (14, 15). To address this, recent efforts have focused on systematizing clinical assessment to better identify patients requiring further evaluation (13, 16).

Motor and sensory impairments in the hands, along with altered gait patterns, are among the most common initial clinical presentations (16). While bladder dysfunction is less frequent, it remains the most common autonomic complaint (16). The sensitivity and specificity of traditional clinical examinations for myelopathy vary depending on the test used. Recent studies have sought to consolidate evidence on the diagnostic utility of neurological exams in myelopathic patients, with Tromner’s sign demonstrating the highest diagnostic accuracy, albeit based on relatively small patient cohorts (13, 17, 18, 19, 20).

### Clinical and radiological findings guiding the decision-making to surgery

Clinical characteristics remain central in predicting surgical outcomes for DCM. Several studies emphasize the importance of disease severity and timing of intervention. Friesen *et al.* (21) reported that patients with moderate myelopathy had significantly better chances of full recovery compared to those with severe disease, particularly when surgery was performed early. Tetreault *et al.* (2) highlighted that decompression within 4 months of symptom onset was associated with improved neurological outcomes, with diminished recovery potential beyond 32 months. Additional data support that preoperative disease severity is a strong predictor: in a 5-year follow-up study of 145 patients undergoing ACCF, those with baseline JOA scores ≤9 were nearly five times more likely to experience limited recovery compared to those with scores >9. Larger cohort studies of patients undergoing laminoplasty also corroborated that higher preoperative JOA scores – and younger age – correlate with better outcomes. Tetreault *et al.* (2) proposed a threshold of mJOA 12, below which reduced improvement is expected (22). Symptom presentation also influences prognosis: neck pain has been consistently associated with postoperative satisfaction and improvement in quality of life (23, 24), while motor symptoms such as gait impairment and hand clumsiness have shown mixed predictive value depending on the outcome measure used (e.g., mJOA vs QoL tools) (25, 26). Importantly, the mJOA score, although widely used, suffers from a ceiling effect in mild cases, limiting its sensitivity to detect meaningful improvement (24). Despite surgical intervention effectively halting progression in most cases, the degree of improvement remains variable. On average, postoperative gains in mJOA score are proportional to the severity of the disease, with average gains of 1.3 points for mild, 2.6 points for moderate and 4.9 points for severe DCM patients (27). However, intra-category results are quite variable, highlighting the need for comprehensive preoperative evaluation that integrates clinical, imaging and electrophysiological parameters to better predict outcomes (22).

#### What is the speed of progression of DCM?

Advancements in knowledge about the natural history of DCM’s are fundamental to guide surgical indications and have thus been selected among the research priorities of the RECODE-DCM project (5). Among the evidence suggesting the appropriateness of nonoperative treatments for mild myelopathy are data suggesting a generally slow progression of myelopathic symptoms, with many patients presenting stationary clinical scenarios for a long time (28). However, it is noteworthy that most studies on natural history are based on the use of the mJOA scale to detect clinical progression; this scale has been associated with various flaws, namely low sensitivity and a ceiling effect (meaning that, for patients at the high end of the scale, relatively large changes in the clinical picture are required to produce a change in score) (24, 29, 30).

More recent studies that took into account multimodal measures of patients’ neurological status and well-being have shown significant rates of clinical deterioration during follow-up (57% at 2.5 years from diagnosis), defining DCM as a disease with a poor prognosis (29).

Among the factors that have been associated with a faster progression are the presence of a circumferential compression (simultaneous anterior and posterior), an increase in range of motion (see the paragraph on spinal alignment below) and the presence of radiculopathy (as already discussed) (5). In particular, in a notorious study (31), the main factors associated with neurological deterioration in nonmyelopathic patients were: symptomatic radiculopathy (>60% in the progressive DCM group versus 26% in the nonprogressive DCM group), MEP/SSEP abnormalities and severity of cord compression on MRI (Fujiwara ≤0.4 and/or cross-sectional area ≤70 mm). A multivariate model based on these risk factors could predict early myelopathy development in more than 80% of cases. Overall, the risk of progression to clinical myelopathy was estimated at 22.6% at 4 years and >50% at 10 years, with rapid progression of DCM (<12 months) in 35.5% of patients. These considerations were confirmed in later works by the same group (32). The presence of spinal cord T2 hypersignal has no clear predictive role (5, 33). In spite of this, in ‘real life’ clinicians seem to attribute significant weight to T2 hypersignal in guiding surgical indications (34).

#### What is the role of MRI findings?

While traditional morphometric measures such as canal diameter, Pavlov’s index or maximum compression area have not shown consistent predictive value in mild DCM (24), MRI findings – particularly signal changes – are critical for both diagnosis and prognosis. A region of intramedullary T2 hyperintensity is seen in the majority of DCM patients (58–85%) and correlates with neurologic impairment. When the hyperintensity is poorly circumscribed, it may reflect acute edema or early Wallerian degeneration, which could be reversible post-surgery. In contrast, sharply demarcated T2 signal changes indicate chronic gliosis and tissue loss, associated with limited potential for recovery. Similarly, hypointensity on T1-weighted images has been linked to worse baseline neurologic status and reflects irreversible injury (22, 32). However, as previously stated, the relationship between spinal cord signal changes and the risk for disease progression is not transparent (5, 33).

**Figure 2:**
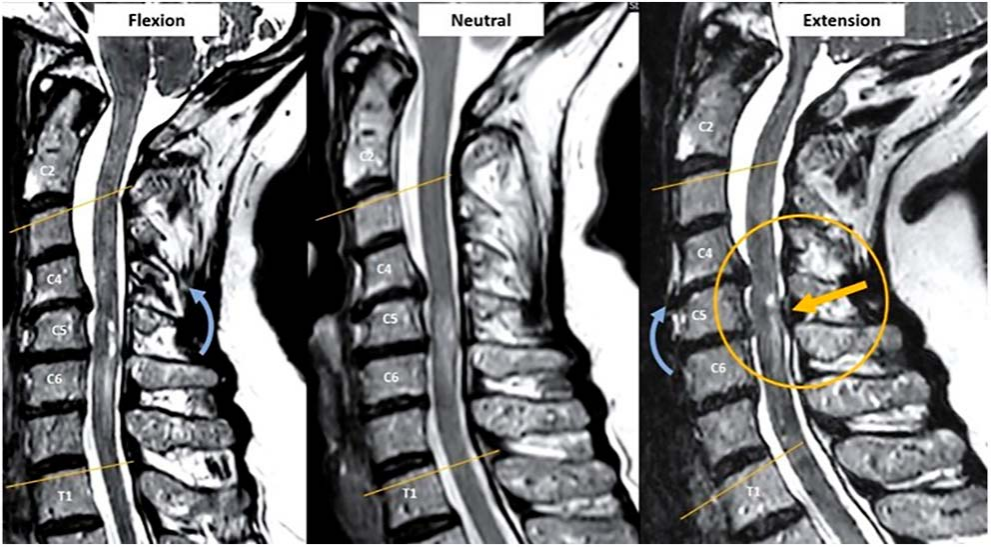
Dynamic MRI revealing severe spinal cord compression in extension at C5-C6, not clearly visible on neutral MRI.

More than traditional MRI, developments in imaging techniques could have a pivotal role in determining the appropriateness and timing of surgical intervention for DCM. Dynamic MRI may be useful to reveal spinal cord compression that is not obvious on neutral MRI (Fig. 2). Indeed, dynamic MRI has shown a better ability to evidence spinal cord signal changes (potentially identifying precocious lesions) and to better define the cranio-caudal extent of cord compression, potentially guiding surgeons to include in the decompression levels that would not be apparent in conventional, neutral MRI (35, 36).

In addition, advanced MRI protocols could help identify the severity of neural damage at earlier stages, before the appearance of overt T2 signal changes, and thus guide surgical indications in patients with mild clinical pictures (7). Moreover, advanced imaging methods could overcome the limitations of traditional clinical scores, such as the mJOA, that can lead to miscategorization of patients due to nonlinearity, subjectivity of assessment and confusion by concomitant disabilities (7). In this respect, cord fractional anisotropy shows a good correlation with severity of the clinical pictures, potentially aiding in clarifying ambiguous presentations. ADC, on the contrary, does not present a clear link with the clinical picture but has shown a promising correlation with postoperative outcomes, potentially representing a viable tool in guiding surgical indications in mild DCM, where precise recommendations lack (37).

**Figure 3:**
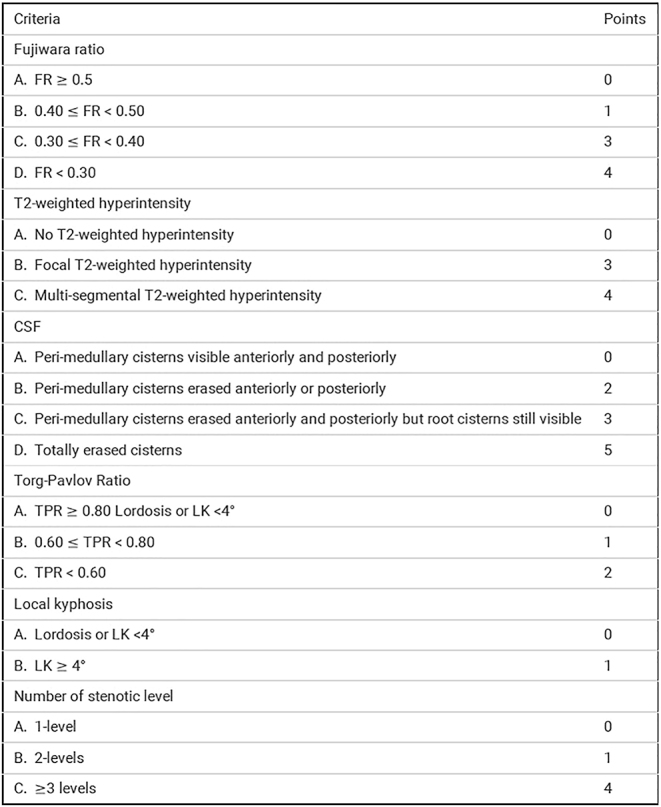
The SIMS is based on six criteria with a maximum of 20 points. Spearman correlation between SIMS and mJOA was calculated as 0.75 (95% CI [−0.823, −0.64]) (Morgado A. The SIMS: severity on imaging myelopathy score. New scoring system of DCM based on MRI (dissertation). (Lyon): Université Claude Bernard – Lyon 1; 2021. 147 p).

In contrast to isolated sign, the multiparametric MRI score, taking into consideration several parameters (as reported by Morgado’s master degree thesis, 2021 – SIMS score, Fig. 3), demonstrated a better correlation of up to 0.75 with the severity of the disease.

### Current treatment guidelines

The most recent international recommendations include the 2017 guidelines jointly published by AOSpine and the Cervical Spine Research Society (CSRS) (1) and the 2019 recommendations from the WFNS Spine Committee (10). The 2017 guidelines, based on the five critical literature reviews conducted by a multidisciplinary group of experts, remain the most authoritative publication to date and have not been updated, as no significant new evidence has emerged to warrant changes. These guidelines strongly recommend surgical intervention for patients with severe (group 5, mJOA: ≤11) and moderate (group 4, mJOA: 12–14) myelopathy, based on moderate-quality evidence. However, for patients with mild clinical myelopathy (group 3, mJOA: 15–17), the recommendations are less clear. The document suggests either surgical intervention or a trial of nonsurgical treatment (‘supervised rehabilitation’), with secondary surgery recommended for those who experience clinical progression and suggested for those who do not improve with rehabilitation. However, these recommendations are weak and based on low-quality evidence. For nonmyelopathic patients with radiological evidence of cord compression, the presence of radiculopathy (group 2) indicates an increased risk of clinical progression. In these cases, either surgical intervention or rehabilitation is suggested, though the supporting evidence is of low quality. In patients without any clinical manifestations (myelopathy or radiculopathy, group 1), observation and counseling are recommended based solely on expert opinion, as no literature supports prophylactic surgery.

The 2019 WFNS recommendations (10) differ from the 2017 guidelines in two key ways. First, for patients with mild myelopathy, they propose that nonoperative management may remain an option even in cases of progressive deterioration, provided the progression is slow. Second, they recommend surgery for patients with radiculopathy and radiological evidence of cord compression, whereas nonsurgical management is only advised if the patient declines surgery. In addition, these guidelines emphasize the importance of obtaining informed consent regarding the risk of neurological deterioration when opting for nonsurgical treatment.

The framework established by these documents allows for clinical judgment in cases of mild myelopathy and nonmyelopathic patients with radiculopathy (groups 2 and 3), where individualized patient assessment is essential. Future research should prioritize this subgroup to better identify patients who would benefit most from early surgical intervention versus those with a more benign course who may be better managed through rehabilitation and nonsurgical strategies.

#### Surgery in mild DCM: when?

Multicenter studies on patients with mild myelopathy have revealed several key findings. First, these patients experience a significantly lower quality of life compared to age-matched healthy individuals. Second, early surgery has been shown to improve quality of life, neurological function and disability measures (38). In addition, early surgical intervention is associated with lifetime gains in quality of life and is considered cost-effective (39). However, surgery-related complications remain a concern, with up to 30% of patients experiencing at least one complication (38).

Other international studies examining ‘real-world’ clinical practices have provided further insights. An international prospective study found that among patients with mild myelopathy, surgery is more frequently offered to those with lower mJOA scores and worse quality of life and disability metrics (40). Factors such as symptom duration, demographics and radiological findings appeared to have less influence on surgical decision-making. However, a large international survey highlighted that spinal alignment and T2 cord hypersignal were among the most influential factors in determining surgical intervention for mildly myelopathic patients (34), despite the lack of strong evidence supporting these criteria (5, 33).

#### What is the role of nonoperative treatments?

In spite of ‘nonoperative treatments’, and especially physical therapy, being offered as an alternative to surgical treatment for mild forms of DCM, there is a paucity of evidence comparing treatments in the literature, with the last systematic review published in 2017 (41). Low-quality evidence exists that for patients with ‘milder’ (mJOA: ≥14) myelopathy, conservative treatment and surgical treatment may have the same effect on post-treatment mJOA and NDI scores. However, the types of conservative treatments adopted are either heterogeneous or non-specified (41, 42). In particular, there is a lack of studies dealing with physical therapy. According to an international survey, only 1/3 of patients with mild myelopathy reported benefits from physical therapy, with efficacy being less likely the more the diagnosis was delayed (43). It is worth mentioning, however, that structured rehabilitation programs may yield meaningful functional improvements not captured by conventional outcome scales such as mJOA.

To date, overall evidence on nonoperative strategies is thus insufficient to influence decisions on the timing of surgery. Research in this area has been identified as one of the RECODE-DCM priorities, potentially allowing for preventing deterioration and delaying or avoiding surgery in patients with mild DCM (44).

### Special considerations

#### OPLL

**Figure 4:**
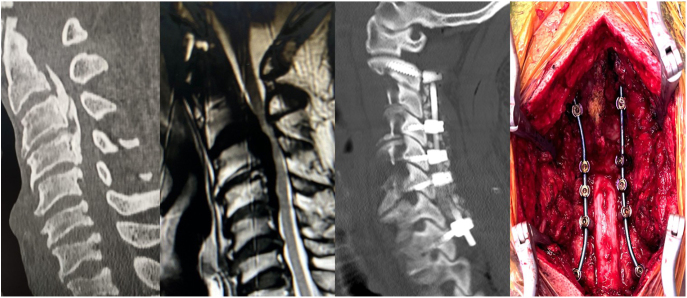
From the left, a cervical CT scan and MRI showing a C3-C4 massive OPLL. A surgical treatment consisting of laminectomy and C2-T1 fixation was performed.

The presence of ossification of the posterior longitudinal ligament, a well-known cause of progressive spinal cord compression, could be a modifier in the choice of the surgical timing (Fig. 4). Although generally, recommendations for OPLL patients have been the same as for non-affected patients (i.e., surgery for moderate/severe clinical myelopathy) (45), a significant portion of surgeons from the Asia-Pacific spine society (reflecting practices where OPLL has a much higher incidence compared to Western countries) performs early or even prophylactic surgery, according to an international survey.

The progressive nature of OPLL is well established. Interestingly, studies on OPLL natural history have documented significantly higher rates of OPLL progression in younger patients (10% annual volume increase in patients between 30 and 49 years vs 4% across all ages), especially in the interrupted variant (46), although this datum is controversial. There is a putative role of increased mobility of the cervical spine in favoring the progression of OPLL (46, 47); this would explain the faster progression in younger patients with discontinuous OPLL and the slower progression rates in older patients, patients with continuous OPLL (acting as a bony bridge) (46) and patients operated on with posterior cervical fusion (48). Moreover, increased cervical mobility has been shown to promote myelopathy development in asymptomatic OPLL patients with canal diameter <14 mm (49).

As compared to ‘classic’ degenerative compression, OPLL may be associated with a greater risk for myelopathic aggravation after minor trauma (42, 50). The weight of such evidence in guiding surgical decisions is however low (27).

Starting from such considerations, and based on retrospective data, some authors have recommended surgery in mildly symptomatic patients if cord compression is significant, based on the good outcomes and fairly low complication rates that can be obtained with proper surgical technique (51). The lack of definitive evidence still leaves room for discussion and further developments.

#### Comorbidities, frailty and advanced age

The role of sociodemographic and health-related factors in outcome prediction is increasingly recognized. Age alone has shown inconsistent correlation with outcomes. While (52) found no association between age and surgical recovery using mJOA or Nurick scores, other large studies (e.g., AO Spine International) reported that patients over 65, despite benefiting from surgery, had lower mJOA and Nurick scores at 24 months. Frailty has emerged as a more meaningful predictor than chronological age, correlating more strongly with mortality, perioperative complications and functional outcomes (22). Contributing factors include increased degenerative burden, more complex surgeries, higher comorbidity burden and overlapping musculoskeletal issues such as osteoarthritis, which may confound neurologic outcome measures. Comorbidities also significantly influence surgical outcomes. While it is easier to balance the weight of comorbidities with the need to stop clinical worsening in patients with moderate and severe disease, in mildly affected patients, considerations on comorbidities can have a greater impact on the choice whether to perform early surgery or to postpone it to when clinical progression becomes evident.

Among cardiovascular comorbidities, hypertension has shown a significant effect of increasing complications and perioperative mortality rates and reducing neurological improvements after surgery, making it more difficult to achieve clinically significant changes in mJOA scores (53, 54). Diabetes has a more nuanced role, with high preoperative HB1Ac values >6.5% and long disease duration (>10 years) decreasing the impact of surgery on neurological recovery; fasting glucose levels seem to be a less important predictor (53). Diabetes, moreover, has a well-known impact on perioperative complication rates (53, 54).

These data suggest that even in patients with a clear surgical indication, a preoperative optimization of the management of medical comorbidities, especially hypertension and diabetes, is advised to improve outcomes.

As for the geriatric population, the decision-making process cannot be exempt from a comprehensive evaluation of factors such as comorbidities, preoperative disease progression and functional status before excluding patients based solely on age. The literature suggests that, with careful management of these risk factors, elderly patients with CSM can actually benefit from surgical intervention.

Studies found that octogenarian/nonagenarian patients with cervical spondylotic myelopathy (CSM) were less likely to be discharged to their homes compared to younger age groups (60–79), although this did not significantly affect other outcomes such as multi-morbidity, prolonged length of stay (LOS), readmission or reoperation rates. While older age is associated with higher risks, the study argues that this should not preclude octogenarians from undergoing surgical interventions such as posterior cervical fusion (PCF), especially when patients are carefully selected.

According to Choi *et al.* (55), laminoplasty emerges as a preferable posterior approach for geriatric patients, offering shorter hospital stays, lower morbidity and fewer complications compared to PCF. The study suggests that older patients often have more extensive spinal disease, making a posterior approach necessary in certain cases. When a posterior approach is required, LP should be prioritized over PCF due to its lower complication rates and cost-effectiveness. Ultimately, while ACDF remains the safest option for geriatric patients, LP is a viable alternative when posterior surgery is needed, minimizing risks associated with PCF.

#### Alignment and deformity

The relationship between cervical spine alignment and development and progression of CSM has been a matter of extensive study and debate. The core principle echoed in the literature is that kyphotic alignment increases tension on the spinal cord, promoting neural degeneration and clinical progression (56). To a certain extent, loss of lordosis is a compensatory mechanism in the first stages of cord compression, as it increases the diameter of the cervical spinal canal. However, at some point this mechanism transforms into a vicious spiral with progressive deformity, increase in cord tension and progression of symptoms. Anyway, there has been a lack of studies focusing on the relationship between static and dynamic alignment and severity and pace of disease progression. Although some studies highlighted a relationship between parameters indicating greater cervical lordosis and range of motion (such as cervical lordosis in maximal flexion, lordosis range of motion, cervical tilt and cervical curvature index) and milder mJOA scores (56, 57), other studies focusing on the comparison between patients with slower disease progression and patients with rapidly progressive CSM found that smaller lordosis in maximal flexion and lordosis ROM could actually predict a more ‘benign’ disease progression. Interestingly, cervical lordosis in resting state did not differ between the groups (58). Data on ‘resting’ cervical lordosis are conflicting, with some evidence of slower disease progression in patients with >29° of cervical lordosis (57).

The role that deformity considerations should have in guiding the timing of surgery is still poorly defined. There is limited data that, among patients with mild myelopathy and cervical spine deformity, a delay in surgery is associated with more complications and reoperation rates (59). In summary, the quality and strength of evidence is too low to formulate any recommendations based on cervical sagittal parameters and deformity.

#### Trauma

**Figure 5:**
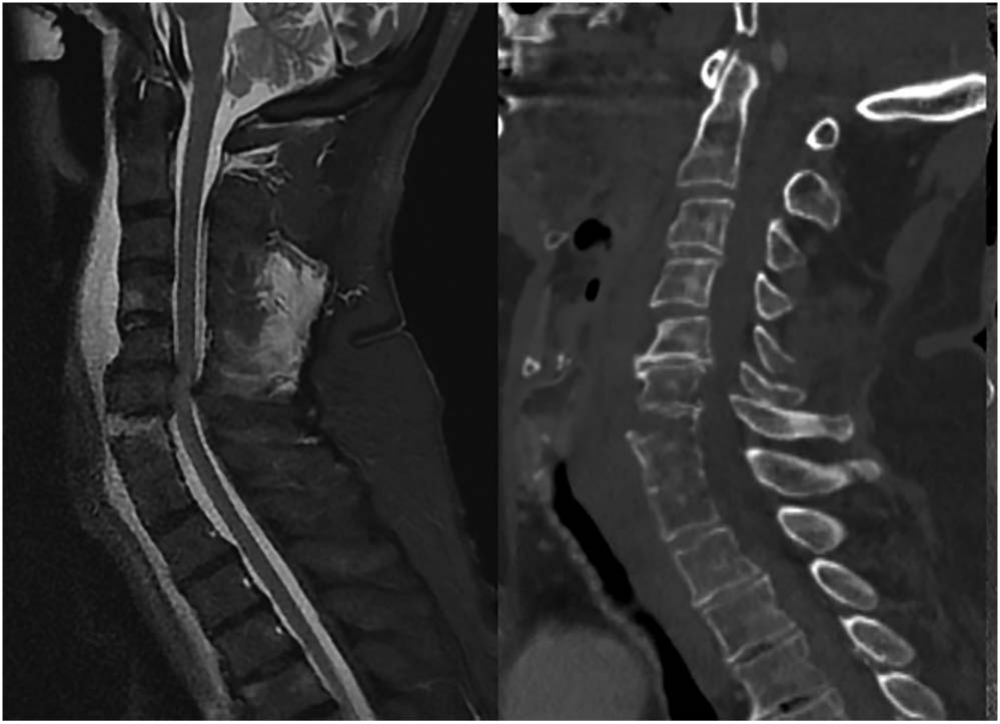
From the left, a cervical spine T2-w MRI and a bone CT, showing a C6-C7 fracture with canal stenosis and medullary compression.

Traumatic events can precipitate neurological deterioration in patients with cervical spinal cord compression, which is associated with an increased risk of developing post-traumatic central cord syndrome (Schneider’s syndrome) (Fig. 5). In spite of the traditional belief that performing acute surgical treatment in this eventuality could lead to further neurological worsening, more recent evidence and recommendations suggest that early surgery (within 24 h from the traumatic event) is actually beneficial and associated with better long-term outcomes (60, 61, 62). Timely intervention can thus be recommended in patients with cervical cord compression that develop acute neurological deterioration following trauma. Spinal cord injury in the context of pre-existing cervical stenosis and absence of instability is reported in the literature as ‘central cord syndrome’. There are few high-quality papers focusing on this group, with confusion between the different scenarios. The role and timing of surgery in this population, in contrast to acute spinal cord injury with a pre-existing normal spine, is still a matter of debate.

Recently, Badhiwala *et al.* performed a prospective multicenter analysis of data derived from three independent institutions. A harmonized dataset combining prospective data from the NACTN, STASCIS and NASCIS III registries included 1,692 patients who underwent surgical decompression for SCI. After applying eligibility criteria, a final study cohort of 186 patients was analyzed – 93 in the early-surgery group (<24H) and 93 in the late-surgery group (≥24H). They reported that early decompression resulted into significant improvement in upper limbs compared to late decompression, with better results observed for patients ASIA C patients. Furthermore, among those with ASIA grade C injuries, early intervention also correlated with improved lower limb motor function at 1-year follow-up, contributing to a greater overall motor score (61).

On the other hand, performing surgery on patients with milder forms of DCM solely based on the risk of aggravation following even minor trauma is not supported by the literature, in view of the low overall incidence of central cord syndrome as compared to the prevalence of paucisymptomatic or asymptomatic cervical cord compression (42).

### Surgery: which one and why

The selection of the surgical approach in DCM is determined by several preoperative factors, including the location and extent of spinal cord compression, cervical alignment, the number of involved levels and morphologic characteristics on imaging. An anterior approach is typically favored in cases of ventral compression, particularly when limited to one or two levels or associated with cervical kyphosis (63). Common anterior procedures include anterior cervical discectomy and fusion (ACDF), anterior cervical corpectomy and fusion (ACCF) and hybrid constructs. Among these, multilevel ACDF is often preferred due to its efficacy in restoring sagittal alignment and reducing postoperative neck pain, with complication rates similar to ACCF in terms of dysphagia, infection and non-union (64). ACCF is better suited for cases involving retrovertebral compression, while hybrid approaches offer comparable neurologic recovery with superior sagittal correction and reduced postoperative discomfort (64, 65). In addition, cervical disc replacement has emerged as a motion-preserving alternative in selected patients, demonstrating equivalent long-term clinical outcomes and complication rates compared to ACDF (66, 67). Posterior approaches are generally selected for multilevel compression, particularly in patients with preserved cervical lordosis. Laminectomy with fusion provides effective decompression and stabilization but is associated with longer operative times, higher costs and greater incidence of complications such as axial neck pain and C5 palsy (68, 69). Laminoplasty, in contrast, preserves motion and posterior elements, leading to better maintenance of cervical range of motion, less disability and lower rates of postoperative kyphosis, though it may be less effective in correcting sagittal imbalance (70, 71). In the context of ossification of the posterior longitudinal ligament (OPLL), surgical planning becomes more complex. When canal occupancy exceeds 60%, anterior decompression is associated with better neurologic outcomes despite increased technical risks (72). Posterior approaches are considered safer in less severe cases of OPLL, although motion-preserving techniques such as laminoplasty have been associated with a significantly faster postoperative progression of ossification (73). Imaging tools such as the modified K-line can aid in determining whether posterior decompression is sufficient; if the anterior compression lies within 4 mm of the K-line, posterior surgery may be inadequate, making an anterior approach preferable (74, 75). Restoring cervical sagittal balance is another critical goal in DCM surgery, as preoperative imbalance is associated with more severe myelopathy and poorer postoperative outcomes. Surgical correction has been linked to improved neurologic recovery, reduced axial pain and better long-term functional scores (76, 77, 78). Emerging posterior techniques such as skip laminectomy and minimally invasive laminoplasty aim to reduce soft tissue damage, preserve biomechanics and minimize postoperative complications, with promising preliminary results (79, 80, 81).

### Future developments

As evident from the previous paragraphs, there is still ample room for developments leading to optimization of treatment decisions and individualizing the timing of surgery based on a multimodal assessment of each patient.

As concerns basic clinical assessment and recognition of the disease, developments in artificial intelligence and machine learning and their application to clinical examination via smartphones appear as promising and accessible strategies to overcome the limitations of ‘traditional’ clinical examination, opening scenarios that could allow, in the near future, to significantly improve awareness, diagnostic capabilities and timely diagnosis (18).

While traditional electrophysiology tools such as motor evoked potentials and somatosensory evoked potentials have been labeled as a cold topic in the DCM research landscape (82), other neurophysiological tools such as contact heat evoked potentials have shown a promising ability to evidence early neural dysfunction in patients with spinal cord compression before the appearance of MRI changes in the spinal cord (8).

Moreover, research on DCM biomarkers is a developing and promising field. While several studies have explored the landscape of CSF markers of myelopathic changes, more recently, studies have focused on circulating RNAs with interesting results (6). Their preliminary findings show a correlation between the expression of certain RNAs (implicated in neural regeneration, damage and inflammation) and the presence of myelopathy. This could provide further guidance in diagnosis and early identification of patients that are undergoing more severe myelopathic changes and would be thus candidate to early surgery.

At the mechanistic level, recent animal studies have shed light on the biological processes driving poor outcomes following delayed intervention (83), demonstrating that ischemia-reperfusion injury initiates a cascade of neuroinflammatory responses – neuronal death, gliosis, chronic pain – that contribute to poor neurological recovery. These findings echo the clinical evidence that delayed surgery results in more permanent damage due to cumulative biological insults. As such, neuroinflammation and ischemia-reperfusion may become targets for adjunct therapies aiming to enhance postoperative recovery (22). In this respect, one of the most discussed agents is riluzole, a neuroprotective agent already in use for the treatment of lateral amyotrophic sclerosis, which could abolish perioperative mechanisms of ischemia-reperfusion injury in DCM (30). Although perioperative administration of riluzole for 6 weeks has not shown significant benefits on mJOA scores in a phase III randomized controlled trial (84), a review of the data using a more integrated evaluation of outcomes has demonstrated that the drug can have significant beneficial effects (85). Preliminary data regarding other agents (cerebrolysin, limaprost alfadex, cilostazol, erythropoietin, G-CSF and Jingshu Keli) have shown promise, but further research and larger studies are needed to validate the evidence (85, 86).

### Summary

**Figure 6:**
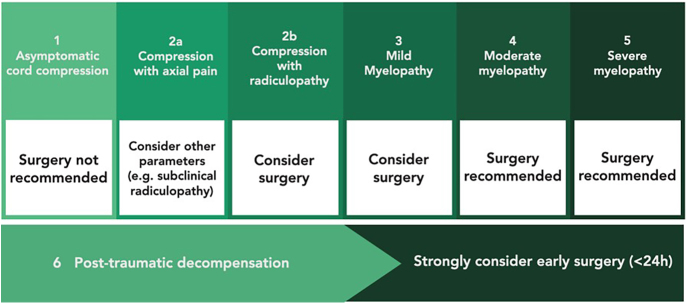
This scheme summarizes our discussion and amplifies the clinical evaluation to seven possible scenarios, where the evidence of medullary compression with axial pain is separated from cases with associated radiculopathy and a traumatic spinal cord injury in the context of spondylotic canal stenosis is taken into account as a different clinical entity.

Figure 6 summarizes our discussion and amplifies the clinical evaluation to seven possible scenarios, where the evidence of medullary compression with axial pain is separated from cases with associated radiculopathy and a traumatic spinal cord injury in spondylotic canal stenosis context is taken into account as a different clinical entity.

## Conclusions

Across the spectrum of DCM, international guidelines consistently recommend surgery as the optimal treatment for patients with moderate to severe disease. For mild cases, a personalized approach is essential, considering neurological status, multimodal imaging findings and overall medical condition. This can allow to find the optimal window for intervention for each patient. Shared decision-making is crucial, ensuring that patients understand both the risks of disease progression and the potential benefits and complications of surgery on quality of life and disability. Among early disease forms, non-myelopathic patients with cervical radiculopathy present a specific challenge, and their increased risk of developing myelopathy makes surgical intervention a reasonable consideration, also taking into account the considerations mentioned earlier. Finally, there is no evidence supporting prophylactic surgery for fully asymptomatic individuals with radiological evidence of cord compression.

## ICMJE Statement of Interest

The authors declare that there is no conflict of interest that could be perceived as prejudicing the impartiality of the work reported.

## Funding Statement

This work did not receive any specific grant from any funding agency in the public, commercial or not-for-profit sector.

## Author contribution statement

CYB conceived the research and supervised the study. MRF and AB conducted the research and elaborated the manuscript. DC and AM contributed to data collecting and editing. All authors contributed to the writing of the manuscript and approved the final version.
